# *Paenibacillus alvei* K165 and *Fusarium oxysporum* F2: Potential Biocontrol Agents against *Phaeomoniella chlamydospora* in Grapevines

**DOI:** 10.3390/plants10020207

**Published:** 2021-01-22

**Authors:** Fedon-Ioannis Gkikas, Alexandros Tako, Danai Gkizi, Christina Lagogianni, Emmanouil A. Markakis, Sotirios E. Tjamos

**Affiliations:** 1 Laboratory of Plant Pathology, Agricultural University of Athens, 75 Iera Odos str., 11855 Athens, Greece; fedonasgk@yahoo.com (F.-I.G.); alexandrosgtako@gmail.com (A.T.); danai_gkizi@aua.gr (D.G.); christinalagogianni@hotmail.gr (C.L.); 2 Laboratory of Mycology, Department of Viticulture, Vegetable Crops, Floriculture and Plant Protection, Hellenic Agricultural Organization Demeter, Mesa Katsampas 71003, Iraklio Crete, Greece; markakis@nagref-her.gr

**Keywords:** biological control, esca disease, lignin, plant defense

## Abstract

In the last two decades grapevine trunk diseases (GTDs) have emerged as the most significant threat for grapevine sustainability worldwide. The tracheomycotic fungus *Phaeomoniella chlamydospora* (Pch) is the predominant GTD-associated species and cannot be controlled with available chemicals. In the present study, we evaluated the effectiveness of two microbial strains (*Paenibacillus alvei* K165 and *Fusarium oxysporum* F2) against Pch in grapevine. In vitro bioassays, performed in a growth culture medium simulating the xylem environment, indicated that F2 decreased Pch growth and sporulation, whereas K165 did not have any effect on Pch growth. *In planta* experiments revealed that root-drench and stem-puncture application of K165 and F2 reduced the endophytic relative DNA amount of Pch by 90% and 82%, respectively, compared to controls. However, wood discoloration, the typical symptom of Pch infection, was not reduced in the F2 treated grapevines. Nevertheless, the F2 treated grapevines harbored higher lignin levels compared to mocks, as it was also done by K165. Therefore, F2 and K165 have the potential to be used as biocontrol agents against Pch in grapevines.

## 1. Introduction

Esca is one of the most significant diseases of grapevine and it has been considered to be as old as the vine cultivation [1,2]. It is spread in all grapevine-growing countries of the World, like France [3], Italy [2], Spain [4], USA [5], Australia [6], Greece [7] and South Africa [8]. Red and white berried cultivars are similarly susceptible to the disease, with the worldwide spread cultivars Sauvignon blanc and Cabernet Sauvignon to be considered as the most susceptible and Pinot blanc and Merlot as the most resistant [9]. Esca is a complex disease mainly associated with the fungi *Phaeomoniella chlamydospora* (Pch), *Phaeoacremonium minimum,* and *Fomitiporia mediterranea* [10]. Among these pathogens, Pch is the most commonly isolated fungus [11]. This species is worldwide spread and has a predominant role in several other grape trunk associated diseases (GTDs) such as ‘Petri disease’ or ‘Grapevine leaf stripe disease’ which affect mostly 1–7 year-old grapevines [2].

*P. chlamydospora* together with *Phaeoacremonium* spp. are primary vascular tissue colonizers in young grapevines, followed by several secondary basidiomycetous invaders of the genera *Fomitiporia*, *Phellinus,* and *Inonotus* [12]. Typical internal symptom in Pch affected vines is the vascular browning; whereas more or less severe symptoms, such as leaf yellowing, interveinal chlorosis, and necrosis, along with stunted growth, decline and entire plant death, may occasionally occur [2]. Besides severe losses in grapevine yield, it may also have a non-negligible effect on wine quality [13]. *P. chlamydospora* penetrates vines primarily through wounds, although root infection can also occur [14]. Due to its air-borne and water-splashed nature, the fungus spreads within vineyards [15], while its long-distance spread is made via the latently infected propagating material [16]. Nowadays, Pch consists a major threat for grapevines in all grapevine-growing countries, since there is no effective disease control by chemical means [1]. In these respects and considering the public pressure for environmentally friendly disease control strategies, it would be advantageous to develop biocontrol based strategies against Pch.

Towards this direction, Yacoub et al. [17] reported the efficacy of *Pythium oligandrum* to reduce the necrosis of grapevine cuttings caused by Pch, due to the triggering of the jasmonate/ethylene dependent plant defense mechanism [18]. In nurseries, it was shown that inoculation of stems with *Trichoderma harzianum* reduced vascular streaking induced by Pch [19]. While, in the vineyard, treating fresh pruning wounds with *Trichoderma atroviride* strain USPP-T1 decreased the incidence of Pch by 77% [20]. In addition, Alfonzo et al. [21] reported the efficacy of *Bacillus subtilis* strain AG1 to inhibit mycelial growth of Pch and *P. aleophilum* due to antibiosis. More recently, Andreolli et al. [22] reported the isolation of soil inhabiting *Pseudomonas* strains antagonistic to Pch; as it was also observed by Niem et al. [23], in the case of grapevine-endophytic *Pseudomonas* strains.

In view of all the above, we examined the efficacy of the biocontrol agents *Paenibacillus alvei* K165 and *Fusarium oxysporum* F2 to protect grapevines against Pch. Both strains have been isolated from *Verticillium dahliae*-suppressive soils and they can protect various herbaceous and woody crops against Verticillium wilt through various modes of action [24,25]. Interestingly, it has been shown that stem injection of F2 in eggplants resulted in reduced *V. dahliae* caused symptoms [26]. As far as the mode of action of F2 is concerned, the use of a transformed F2 strain with the enhanced green fluorescent protein reporter gene revealed that competition for space or nutrients is the main mode of action of F2 against pathogens [26]. Whereas, the plant protective activity of K165 has been attributed to the triggering of plant defense mechanisms via an SA-dependent pathway [27]. Therefore, knowing the plant protective activity, mode of action, and application methodologies of K165 and F2, we investigated the plant protective activity of both BCAs in grapevines of cv Soultanina (*Vitis vinifera* L.) against Pch.

## 2. Results

### 2.1. Evaluation of the Plant Protective Activity of K165 and F2 against Phaeomoniella chlamydospora

**Figure 1:**
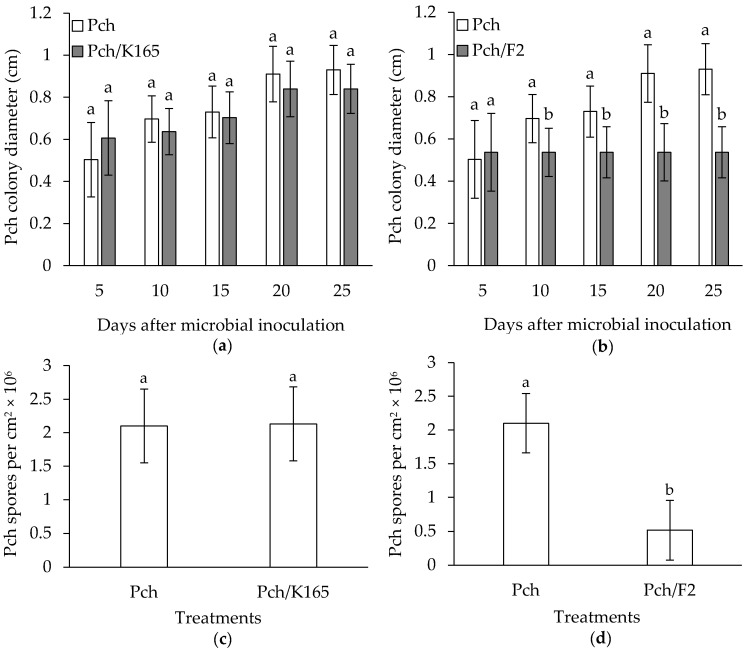
In vitro growth (**a**,**b**) and sporulation (**c**,**d**) of *Phaeomoniella chlamydospora* (Pch) in dual culture with *Paenibacillus alvei* K165 (Pch/K165) or *Fusarium oxysporum* F2 (Pch/F2) in agar medium simulating the xylem environment. The sporulation of Pch was assessed at 25 days after inoculation. The columns represent the means of three biological repeats (*n* = 3) (with three plates per treatment and repeat). Columns (at each day for the in vitro growth experiment) with different letters are significantly different from each other, according to a t-test (*p* < 0.05). The vertical bars indicate the standard difference value.

The in vitro evaluation of K165 and F2 against Pch was performed on a growth medium simulating the xylem environment (SXM) [28]. It was shown that the growth and sporulation of Pch were not inhibited significantly by K165 (Figure 1a,c); therefore, K165 does not produce toxic compounds for Pch in the SXM growth medium. On the other hand, F2 reduced the growth of Pch by 43% and the sporulation by 90% on the SXM growth medium, at 28 dpi (Figure 1b,d). It is worth mentioning that F2 inhibited completely the growth of Pch after 5 dpi. Having had those results, we examined whether K165 can protect grapevines of the susceptible to Pch cultivar Soultanina (syn. Sultana, Lady de Coverly and Thompson Seedless) [29], as a soil drenching inoculant by triggering ISR against the pathogen; whereas, F2 was injected in the trunk in order to examine its direct action against Pch, *in planta*.

**Figure 2:**
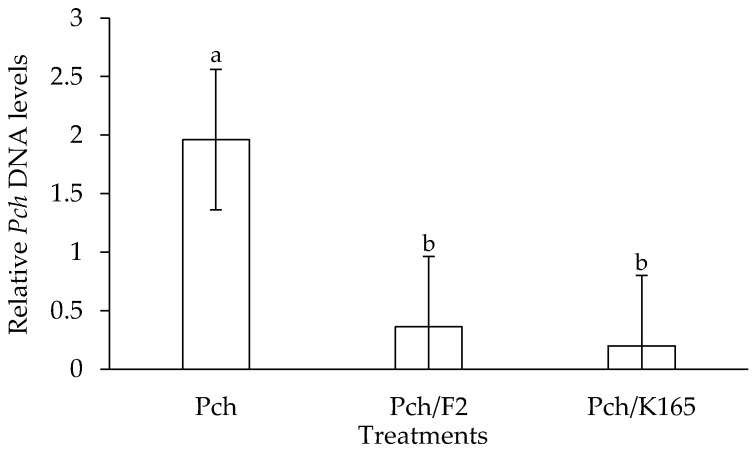
Relative quantification of *Phaeomoniella chlamydospora* DNA levels in the *Paenibacillus alvei* K165 (Pch/K165), *Fusarium oxysporum* F2 (Pch/F2) and control (Pch) treated grapevines. Fungal DNA levels were estimated by qPCR using total DNA isolated from grapevines at 90 dpi. The columns represent the means of three biological repeats (*n* = 3) (with eight grapevines per treatment and repeat). Columns with different letters are significantly different (*p* < 0.05) according to LSD test. The vertical bars indicate the standard difference value.

**Figure 3:**
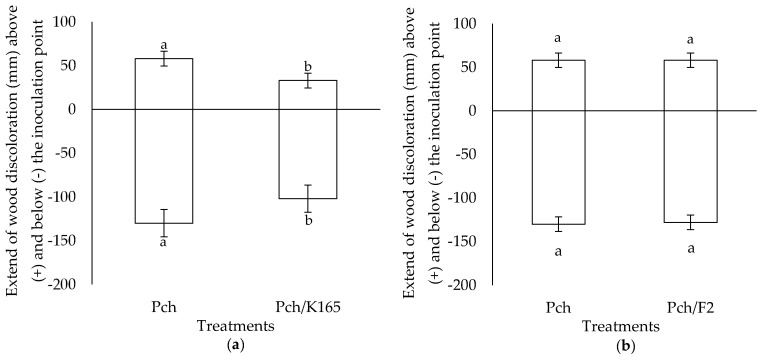
Extent of the wood discoloration above and below the *Phaeomoniella chlamydospora* inoculation point in the *Paenibacillus alvei* K165 (Pch/K165) (**a**), *Fusarium oxysporum* F2 (Pch/F2) (**b**), and control (Pch) treated grapevines. The columns represent the means of three biological repeats (*n* = 3) (with eight grapevines per treatment and repeat). At each site (above or below the inoculation point), columns with different letters are significantly different from each other, according to a t-test (*p* < 0.05). The vertical bars indicate the standard difference value.

The *in planta* experiments revealed that K165 can reduce the relative amounts of the pathogen along with the symptoms of the disease in the grapevine trunk. In particular, the qPCR results indicated that K165 reduced by 90% the relative DNA amount of Pch compared to controls (Figure 2). In addition, the wood discoloration length above and below the Pch inoculation point in the K165 treated vines was significantly lower compared to controls (Figure 3a). Therefore, the application of K165 by root drenching reduced the disease symptoms of Pch in grapevine trunk and the relative DNA amount of the pathogen. Furthermore, the analysis of the K165 population revealed that it remained spatially separated from the pathogen, since it was not detected inside the trunk. On the other hand, the rhizosphere population of K165 was 3 × 10^5^ cfu/g at 90 dpi, indicating a successful colonization of the rhizosphere. Therefore, a priming effect may hide behind the biocontrol activity of K165 against Pch, as it is also suggested by the in vitro absence of deleterious compounds for the pathogen. 

In the case of the F2 treatment, a reduction in the length of the discoloration inside the trunk was not observed, compared to the controls (Figure 3b). However, the relative DNA amount of Pch in the F2 treated grapevines was reduced by 82% compared to controls (Figure 2); this observation may lie on the colonization of vascular tissues by F2, as it was successfully reisolated from xylem vessels at 90 days after Pch inoculation.

### 2.2. Lignin Quantification

The suggestion that K165 may have a priming effect on grapevines led to the idea of examining the lignin levels of Pch infected grapevines upon K165 application. Lignin is a well-studied defense polymer that forms a physical barrier to inhibit pathogen invasion and prevents the diffusion of toxins produced by pathogens [30].

**Figure 4:**
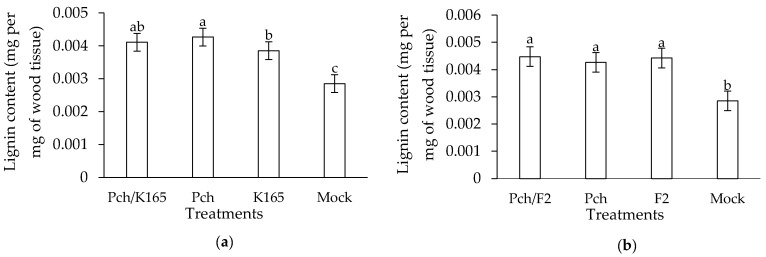
Lignin levels in the *Paenibacillus alvei* K165 (**a**), *Fusarium oxysporum* F2 (**b**), and control treated vines inoculated (Pch/K165, Pch/F2, Pch) or not (K165, F2, Mock) with *Phaeomoniella chlamydospora* (Pch), at 90 days post-inoculation. The columns represent the means of three biological repeats (*n* = 3) (with eight grapevines per genotype and repeat). Columns with different letters are significantly different (*p* < 0.05) according to LSD test. The vertical bars indicate the standard difference value.

The application of K165 in the grapevines, as a root drenching inoculum, resulted in increased lignin levels in the trunk by 25%, compared to mocks (Figure 4a). Similarly, the injection of F2 in the trunk increased the concentration of lignin by 73% compared to mocks (Figure 4b). Therefore, the F2 treated grapevines harbored higher lignin levels than the K165- and mock-treated plants. Upon pathogen inoculation, the lignin levels increased in the K165 and the control treatment, by 62% and 74%, respectively, compared to mocks. On the other hand, the lignin levels in the F2 treated grapevines were not affected by pathogen inoculation, since a significant difference was not observed between the pathogen treated and non Pch treated F2 injected grapevines.

## 3. Discussion

Nowadays, the use of biocontrol agents is an appealing disease management strategy since it possesses fewer threats for the environment than chemicals and can be effective against pathogens inaccessible to fungicides or pathogens prone to developing fungicide resistance. For these reasons, we evaluated the efficacy of a bacterial (K165) and a fungal strain (F2), with proven suppressive activity against other vascular diseases, to protect grapevines against Pch, the predominant member of several GTDs.

Our results revealed that strains K165 and F2 reduce the relative DNA levels of Pch in grapevines of the susceptible cv Soultanina. In the case of K165, we also observed a decrease in the length of the wood discoloration in the trunk compared to controls. The dark-brown to black discoloration of the xylem is considered as the most consistent symptom of Pch infection [31,32]. Wood discoloration, as a brown wood streaking, is caused by the oxidation and polymerization of phenolic compounds, due to the action of plant produced phenolases upon pathogen infection [33]. Previous studies have reported the reduction in wood streaking extent in grapevines due to the presence of fungal biocontrol agents [34,35] and it was mainly attributed to antagonism for space and/or nutrients. However, in our study the fungal biocontrol agent F2 did not reduce wood discoloration. Therefore, F2 may reduce the sporulation or the hyphae mass of the fungus but not its spread inside the trunk. Indeed, the extent of wood streaking may not be correlated with the abundance of the pathogen, as it has been reported in previous studies [2,11]. Like in other studies, we did not observe foliar symptoms caused by Pch since the pathogen may take several months/years to cause foliar symptoms [36].

The biocontrol potential of *Paenibacillus* sp. against Pch has been reported for the first time by Haidar et al. [37], who isolated a *Paenibacillus* sp. strain from Bordeaux vineyards. This strain reduced wood discoloration caused by Pch and triggered the expression of a number of defense-related genes in grapevines of cultivar Cabernet Sauvignon. In the same study, the *Paenibacillus* strain inhibited the growth of Pch in vitro through antibiosis and volatile organic compounds [38]. On the contrary, our results suggest that K165 does not produce secondary metabolites with antimicrobial activity against Pch *in vitro*. However, K165 may produce toxic compounds against Pch under soil condition, since soil parameters like oxygen, temperature and iron availability affect antibiotic production by microorganisms [39].

Lignin is a major component of the cell wall of vascular plants and constitutes the first line of defense against successful penetration of invasive pathogens, like Pch [30]. Lignification is a key process for the cell wall to become more resistant to mechanical pressure exerted by fungal appressoria and less vulnerable to cell wall-degrading enzymes. It has been reported that K165 increases lignin levels when applied to *Arabidopsis thaliana*, conferring resistance against the vascular pathogen *V. dahliae* [40]. Indeed, the application of K165 and to a higher degree of F2 resulted in increased lignin levels in the wood tissues compared to the mocks. Therefore, the predisposition of lignin in the K165- and F2-treated grapevines may retard the spread of Pch and its toxins/effectors in tissues. Previous studies have shown that Pch secretes two pentaketides [41,42,43] and an a-glucan pullulan [42] with phytotoxic properties [44] and also polypeptides that interfere with the plant cell metabolism [41]. It was also postulated that the secreted polypeptides might trigger plant defense elicitation processes as shown in data demonstrating the activation of phenylalanine ammonia-lyase [45], which participates in the synthesis of lignin. Indeed, our data showed that the lignin levels increased in the pathogen inoculated grapevines compared to mocks. However, the control plants (Pch inoculated) had similar lignin levels to K165/Pch and F2/Pch treated plants; therefore, the simultaneous presence of the biocontrol agents and the pathogen did not have a cumulative effect on lignin concentration.

Our study shows the potential of a nonpathogenic *Fusarium* and *Paenibacillus* strain to reduce the biomass of Pch in the wood of the susceptible cv Soultanina and also trigger the accumulation of lignin in the trunk. Our experimental data along with data of previous studies suggest that F2 and K165 may protect plants against a variety of vascular pathogens which are inaccessible to chemicals due to their life cycle. Having had the present results, it is intriguing to investigate in a future study the coinoculation biocontrol effect of K165 and F2 against Pch in grapevines.

## 4. Materials and Methods

### 4.1. Plant Material

Canes of cv Soultanina (*Vitis vinifera* L.), ca. 1.0 cm diameter and 50 cm length, were cut from the middle of 1 year-old shoots of pathogen-free mother plants (Bakasietas Vine Nursery, Leontio, Corinthia, Greece) in late February, dipped in rooting hormone solution (indole butyric acid) and planted in pots (3.5 L) containing a mixture of perlite and peat (1:1, *v/v*). The rooted cuttings were maintained in a growth chamber at 27 ± 2 °C with a 16 h light/dark cycle and 80% relative humidity, for 5 weeks; then, they were transferred to the experimental greenhouse (20 ± 5 °C) and in late April were transplanted in pots (5 L) containing pasteurized soil substrate (Potground, Klasmann, Deilmann, Germany).

### 4.2. Microbial Preparation

A Pch strain (code A5LP1), isolated from a symptomatic vine of cv Romeiko [29], was used in the in vitro and *in planta* experiments. The fungus was grown on potato dextrose agar (PDA, Merck, Darmstadt, Germany) Petri dishes at 24 °C for 15 days. For the *in planta* experiments, the inoculum was prepared by flooding the cultures with sterilized distilled water and harvesting the spores with filtration through three layers of sterilized cheesecloth. The concentration of the spore suspension was adjusted to 2.5 × 10^6^ spores mL^−1^.

The K165, rifampicin resistant mutant [25], and F2, hygromycin B resistant mutant [26], strains were grown in liquid culture of nutrient broth amended with glycerol (NG) and sucrose sodium nitrate (SSN), respectively, in an orbital incubator at 180 rpm, 28 °C for 18 h. The K165 and F2 suspensions were centrifuged at 5000 g for 10 min and resuspended in 50 mM phosphate buffer, pH 7.02, giving a concentration of 10^7^ cfu or spores mL^−1^.

### 4.3. In Vitro Activity of K165 and F2 against Phaeomoniella chlamydospora

The in vitro tests for the F2 and K165 antagonism against Pch were performed according to Reddy and Patrick [43]. In brief, F2 and K165 were placed 2.5 cm from the edge of a Petri dish (9 cm diameter) containing SXM supplemented with agar (15 g L^−1^) [28] and a disc (5 mm diameter) of Pch taken from the edge of an actively growing colony on PDA was then placed in the dish, 2.5 cm from the edge on the opposite to F2 or K165 side. The dishes were placed in the dark at 24 °C and the diameter of the Pch culture was recorded at 5-day-intervals for up to 25 days. At the end of the experiment (25 days after inoculation), we assessed the sporulation of Pch in each treatment. For this purpose, we cut the edges of the actively grown Pch with a cork borer (5 mm diameter) and vortexed them three times, for 30 s each time, in sterilized distilled H_2_O. The sporulation of Pch was assessed under a light microscope by using a hemocytometer and expressed as spores per cm^2^. The experiment was conducted with a completely randomized design with three replications of each treatment and repeated thrice. Therefore, the statistical replicate unit was the group of three technical replicate dishes and the number of replicates for statistical comparisons was *n* = 3.

### 4.4. In Planta Bioassays to Evaluate the Activity of K165 and F2 against Phaeomoniella chlamydospora

Sixteen grapevines cv Soultanina grown in plastic pots containing 3 L of pasteurized soil (Potground, Klasmann, Deilmann, Germany) were either root drenched with K165 at a rate of 500 mL (10^7^ cfu ml^−1^) per pot or stem inoculated with 50 μL spore suspension of F2 (2.5 × 10^6^ conidia mL^−1^) by making a 3.0 × 7.0 mm hole (at a 45-degree angle) in the trunk. The wound was sealed with cellophane membrane and covered with adhesive paper tape. After 5 days, eight K165 and F2 treated grapevines were stem inoculated with Pch (50 μL of 2.5 × 10^6^ spores mL^−1^ per vine), along with eight non K165 or F2 treated grapevines (controls), following the same F2 application methodology. Nonpathogen inoculated K165 and F2 treated grapevines served as negative controls. Eight additional grapevines were mock inoculated. Vines were maintained for 3 months (May, June, and July) under greenhouse conditions with controlled temperature conditions (25 °C). Eight plants were used per treatment (Pch, Pch/F2, Pch/K165, F2, K165, Mock) and the experiment was replicated thrice (24 plants per treatment in total). The statistical replicate unit was the group of the eight technical replicate plants and the number of replicates for statistical comparisons was *n* = 3.

### 4.5. Disease Assessment

At 90 days after Pch inoculation, we evaluated the wood discoloration in each treatment and grapevine, since this is the most consistent symptom associated with Pch infection [33,34,35]. For this purpose, we performed longitudinal and transverse sections of trunks to measure the extension of vascular tissue discoloration above and below the inoculation point. To verify the presence of Pch in vascular tissues of vines, the trunks of three vines per treatment and replication were randomly selected. The trunks were surface sterilized and xylem chips were aseptically removed and placed onto acidified PDA in Petri dishes. Dishes were placed at 22 °C in the dark for 3 weeks. The emerging fungal colonies were examined under a light microscope and identified as Pch according to their morphological characteristics.

### 4.6. Colonization Capacity of K165 and F2 in Vines

The trunk colonization capacity of F2 and K165 was evaluated by following the methodology of Pch isolation, at the end of the *in planta* experiment (90 days after Pch inoculation). The xylem chips were placed onto PDA plates amended with hygromycin B (75 μg mL^−1^) and rifampicin (100 μg mL^−1^) and placed at 28 °C in the dark for 3 days. To estimate the rhizosphere K165 population, 0.5 g rhizosphere soil was collected and shaken for 45 min in 50 mM phosphate buffer (pH 7.02) containing Tween 20 (0.02%). The suspension was plated onto PDA supplemented with rifampicin (100 μg mL^−1^). After incubation at 30 °C for 48 h, the number of K165 cfu g^−1^ of rhizosphere soil was determined.

### 4.7. qPCR Quantification of Phaeomoniella chlamydospora

At the end of the *in planta* experiment, we also evaluated the relative DNA levels of Pch in the grapevines of the different treatments (Pch, Pch/F2, Pch/K165). The vascular tissue (cut at 20 cm below and 10 cm above the Pch inoculation point) of the eight trunks per treatment was pooled in one group and ground to a fine powder, using an autoclaved mortar and pestle in the presence of liquid nitrogen.

Total DNA was isolated according to Dellaporta et al. [44] and the concentration was estimated by using a NanoDrop UV spectrophotometry. qPCR assays for the quantification of Pch were conducted by using the pair of primers 5′-CTCCAACCCTTTGTTTATC-3′ and 5′-TGAAAGTTGATATGGACCC-3′ [45]. qPCRs were performed in an Applied Biosystems StepOnePlus thermocycler and for the amplification reactions, FastGene IC Green qPCR universal mix (NIPPON Genetics EUROPE GmbH) was used. The results were analyzed with StepOne v.2.3 qPCR software. For sample calibration, EF1γ (XM_003633372.1) of grapevine was targeted by using the primer pair 5′-GAAGGTTGACCTCTCGGATG-3′ and 5′-AGAGCCTCTCCCTCAAAAGG-3′ [46]. PCR cycling started with an initial step of denaturation at 95 °C for 2 min, followed by 40 cycles of 95 °C for 5 s and 60 °C for 30 s. Three biological repeats were conducted with eight plants per treatment and repeat, therefore, the number of replicates for statistical comparisons was *n* = 3.

### 4.8. Lignin Quantification

As we did in the case of Section 4.7, the vascular tissue (cut at 20 cm below and 10 cm above the Pch inoculation point, or at the same distance for the nonpathogen inoculated treatments) of the eight trunks per treatment (Mock-inoculated, K165, F2, Pch, K165/Pch, F2/Pch) was pooled in one group and used for lignin extraction according to Schenk and Schikora [47]. In brief, the soluble phenolics were extracted with 80% methanol and the cell wall-bound phenolics were hydrolyzed (alkaline hydrolysis) and dissolved in ethyl acetate. Lignin was extracted by binding to the lingo-thioglycolic acid complex. The lignin complex was dissolved in NaOH and the absorbance was measured at 340 nm. A standard curve for lignin was generated, by using lignin, alkali (Sigma-Aldrich-370959). Three biological repeats were conducted with eight plants per treatment and repeat, therefore, the number of replicates for statistical comparisons was *n* = 3.

### 4.9. Statistics

The experimental data of the in vitro tests and wood discoloration were analyzed by carrying out a two-sample t test (*p* < 0.05). For the data of Pch qPCR and lignin quantification analysis of variance (ANOVA) was applied. When a significant (*p* < 0.05) F test was obtained for treatments, data were subjected to means separation by least significant difference (LSD) test.

## Data Availability

The data presented in this study are available on request from the corresponding author.
